# Enhanced Vaccine-Induced CD8^+^ T Cell Responses to Malaria Antigen ME-TRAP by Fusion to MHC Class II Invariant Chain

**DOI:** 10.1371/journal.pone.0100538

**Published:** 2014-06-19

**Authors:** Alexandra J. Spencer, Matthew G. Cottingham, Jennifer A. Jenks, Rhea J. Longley, Stefania Capone, Stefano Colloca, Antonella Folgori, Riccardo Cortese, Alfredo Nicosia, Migena Bregu, Adrian V. S. Hill

**Affiliations:** 1 The Jenner Institute, University of Oxford, Oxford, United Kingdom; 2 Okairos, Rome, Italy; 3 Okairos AG, c/o OBC Suisse AG, Basel, Switzerland; 4 CEINGE, Naples, Italy; 5 Department of Molecular Medicine and Medical Biotechnology, University of Naples Federico II, Naples, Italy; Federal University of São Paulo, Brazil

## Abstract

The orthodox role of the invariant chain (CD74; Ii) is in antigen presentation to CD4^+^ T cells, but enhanced CD8^+^ T cells responses have been reported after vaccination with vectored viral vaccines encoding a fusion of Ii to the antigen of interest. In this study we assessed whether fusion of the malarial antigen, ME-TRAP, to Ii could increase the vaccine-induced CD8^+^ T cell response. Following single or heterologous prime-boost vaccination of mice with a recombinant chimpanzee adenovirus vector, ChAd63, or recombinant modified vaccinia virus Ankara (MVA), higher frequencies of antigen-specific CD4^+^ and CD8^+^ T cells were observed, with the largest increases observed following a ChAd63-MVA heterologous prime-boost regimen. Studies in non-human primates confirmed the ability of Ii-fusion to augment the T cell response, where a 4-fold increase was maintained up to 11 weeks after the MVA boost. Of the numerous different approaches explored to increase vectored vaccine induced immunogenicity over the years, fusion to the invariant chain showed a consistent enhancement in CD8^+^ T cell responses across different animal species and may therefore find application in the development of vaccines against human malaria and other diseases where high levels of cell-mediated immunity are required.

## Introduction

ME-TRAP is an antigenic construct comprising full-length *Plasmodium falciparum* TRAP (thrombospodin related adhesion protein) fused to ME, a string of 20 malarial T- and B-cell epitopes [1]. Heterologous prime-boost immunization of healthy adults with vectored vaccines encoding ME-TRAP delayed or prevented parasitaemia in a proportion of volunteers challenged with *P. falciparum* infection in three phase 2a clinical efficacy trials [1]–[3]. As new viral vectors have been developed and incorporated into optimized heterologous prime-boost regimens, the mean frequencies of T cells induced against ME-TRAP in humans have also increased [4], from tens of IFN-γ spot forming cells per million peripheral blood mononuclear cells (SFC/10^6^ PBMCs) using DNA plasmid priming and boosting with recombinant modified vaccinia virus Ankara (MVA), to hundreds of SFC/10^6^ PBMCs using priming with recombinant fowlpox virus (strain FP9) and boosting with MVA, and most recently to thousands of SFC/10^6^ PBMCs using priming with a recombinant chimpanzee adenovirus vector, ChAd63 [5], and boosting with MVA [6]. Since there is strong evidence that increased cellular immune responses against ME-TRAP correlate with increased protection against *P. falciparum* challenge [7]–[9], there is a need to improve the immunogenicity of ME-TRAP-expressing viral vectored vaccines even further to achieve clearly deployable levels of vaccine efficacy.

One approach to augment vaccine-induced T cell responses is genetic fusion of the antigen of interest to CD74, the MHC class II invariant chain (Ii). The now well-known, canonical role of Ii in the MHC class II antigen presentation pathway [10], [11] led several researchers to exploit genetic fusion of antigens to Ii as a means of enhancing antigen presentation to CD4^+^ T cells [12]–[15]. But Mary Collins’ laboratory at University College London, UK, was (to our knowledge) the first to describe an unexpected, additional effect on CD8^+^ T cell induction, using immunization with a lentiviral vector expressing ovalbumin fused to Ii (Ii-OVA) [16]. Subsequently, five reports from the University of Copenhagen [17]–[21] have documented enhanced induction of CD8^+^ T cell responses by human adenovirus 5 (HAdV-5) and plasmid DNA vectors expressing Ii-fused antigens.

Vaccination with the glycoprotein of lymphocytic choriomeningitis virus (LCMV-GP), expressed by a HAdV-5 vector [20] or a DNA plasmid [19], was shown to elicit higher frequencies of antigen-specific IFN-γ^+^ CD8^+^ T cells and enhanced *in vivo* proliferation of adoptively transferred CD8^+^ T cells when fused to Ii. HAdV-5 vectored vaccines encoding the Ii-fused antigen conferred improved protection in an LCMV challenge model and in a tumour challenge model using melanoma cells expressing LCMV-GP [18], consistent with prior independent results using lentivirally-delivered Ii-OVA and EG7-OVA tumour challenge cells [16]. Similarly, fusion of the NS3 protein of hepatitis C virus to Ii accelerated and augmented IFN-γ^+^ CD8^+^ T cell responses following vaccination with a HAdV-5 vector encoding this antigen, with no significant difference in cellular phenotype, as assessed by multi-parameter flow cytometry. The reduction in viral titre after challenge with vaccinia virus expressing NS3 was enhanced in an IFN-γ-dependent manner [17]. More recently vaccination with HAdV-5 vector expressing the GP-Ii fusion was shown to protect mice from intracellular bacteria using a *Listeria monocytogenes* challenge model [21].

Genetic fusion of a transgenic antigen to Ii may augment CD8^+^ T cell immunogenicity via enhanced antigen presentation on MHC class I. Bone-marrow derived dendritic cells transduced with vectors expressing Ii-fused antigen have been reported to direct greater antigen-specific CD8^+^ T cell proliferation *in vitro*, without any apparent difference in levels of cell surface costimulatory molecules or total MHC class I [20]. Furthermore, CD4^+^ T cell help is not required for this *in vitro* effect nor for enhancement of HAdV-5 vector-induced CD8^+^ T cell responses *in vivo* [22]. The mechanism of the effect of Ii on MHC class I presentation of a fused antigen remains unclear, mainly due to the large number of functional domains of Ii that could be responsible [20]. Nevertheless, the available evidence supports potential progression of these research findings into an appropriate clinical setting, such as a liver-stage malaria vaccine known to elicit protective human CD8^+^ T cell responses against *P. falciparum*.

Concerns over the safety of the HAdV-5 vectors used to prevent HIV-1 infection, particularly in individuals with high levels of antibodies to this vector [23] have led to increased interest in the use of chimpanzee adenoviral vectors as vaccines, which encounter far lower levels of anti-vector immunity in human populations. Moreover, T cells induced by adenoviral vectors can be substantially enhanced by use of a MVA vector as a heterologous boost [6], [24], but it is unknown whether use of Ii fusions in MVA could enhance immunogenicity. Here, we report that recombinant ChAd63 and MVA vectors with a transgene encoding Ii-ME-TRAP fusion proteins induce higher frequencies of antigen specific CD8^+^ T cell responses in mice and macaques than those expressing the unmodified ME-TRAP protein. This finding may have potential utility in improving the protective efficacy of viral vectored vaccines against malaria in humans.

## Materials and Methods

### Design of Ii-ME-TRAP Fusion Protein

The ME-TRAP antigen construct comprises a human codon-optimized multi-epitope string (ME) fused to the native *P. falciparum* T9/96 strain cDNA sequence encoding TRAP [1]. Also known as sporozoites surface protein 2 [25], TRAP is a type Ia membrane protein with a predicted N-terminal signal peptide, a large ectodomain, a transmembrane domain, and a short cytoplasmic C-terminal domain. This topology is compatible with fusion of the N-terminus of ME-TRAP to the C-terminus of Ii, since the latter is a type II membrane protein with a short cytoplasmic N-terminal domain. In order to prevent signal peptidase cleavage of the TRAP signal peptide, which (if it were to occur) would be predicted to result in hydrolysis of the peptide bond linking the antigen to Ii, nucleotides 1–75 of the TRAP open reading frame (ORF), which encodes a predicted signal peptide, were deleted from ME-TRAP in the versions fused to Ii. A mixture of gene synthesis and conventional cloning was used to make in-frame fusions of this modified ORF to synthetic ORFs (optimized to human codon usage) encoding the human (NCBI RefSeq NP_004346.1; isoform b) or mouse (NCBI RefSeq NP_034675.1; isoform 2) CD74 proteins, respectively.

### Construction of Recombinant Adenovirus Vectors and Recombinant MVA

The above chimeric ORFs encoding ME-TRAP fused to mouse Ii (mIi-ME-TRAP) or to human Ii (hIi-ME-TRAP) were sub-cloned into a transgene expression cassette comprising a modified human cytomegalovirus major immediate early promoter (CMV promoter) with tetracycline operator (TetO) sites [26]. The cassettes were inserted into the E1 locus of an E1/E3-deleted and E4-modified genomic clone of ChAd63 [5], using site-specific recombination, as previously described [26]. The pre-existing comparator construct, ChAd63.ME-TRAP, lacks TetO sites in the CMV promoter (which enable repression of transgene expression during viral production in 293 cells expressing the tetracycline repressor (TetR) protein), and was generated by recombination in BJ5183 cells [26], but is otherwise identical. The viruses (referred to as ChAd63.hIi-ME-TRAP and ChAd63.mIi-ME-TRAP) were then rescued and propagated in 293 or 293-TetR cells, purified by CsCl gradient ultracentrifugation and titred as previously described [27]. Doses for vaccination were based on infectious units (iu), since these, and not viral particles (vp), determine immunogenicity [27]. ChAd63 particle-to-infectious-unit (P:I) ratios were in the range 50–120.

The ORF encoding hIi-ME-TRAP was sub-cloned into a orthopoxviral expression plasmid to place it under control of the vaccinia virus p7.5 promoter and the cassette was introduced into the thymidine kinase (TK) locus of MVA by recombination in transfected and infected chick embryo fibroblast (CEF) cells followed by transient-dominant selection with a GFP marker gene. The resulting viral recombinant, MVA.hIi-ME-TRAP, was plaque-purified and amplified in CEFs, purified over sucrose cushions and titred twice in duplicate in CEFs by an immunostained plaque assay, according to standard methods. The identity and purity of the isolate was verified by PCR. The comparator virus, MVA.ME-TRAP, has a *LacZ* marker gene, but is otherwise identical in design and was purified and titred similarly.

### Ethics Statement

Mice were used in accordance with the UK Animals (Scientific Procedures) Act under project license numbers 30/2414 or 30/2889 granted by the UK Home Office. All mice were humanely sacrificed at the end of the experiment by a Schedule 1 procedure as defined by the UK Home Office.

Ethical approval for use of male rhesus macaques was granted by the University of Wisconsin-Madison IACUC (termed Animal Care and Use Committee) and was assigned protocol number g00677. The Wisconsin National Primate Research Center (WNPRC) executed the study to honor the fee-for service agreement between the University of Wisconsin, USA and the University of Oxford, UK. Processing of blood samples, fresh enzyme linked-immunospot (ELISpot) and fresh intracellular cytokine staining (ICS) were performed at the University of Wisconsin, with serum and frozen PBMCs shipped to Oxford for additional immunological investigations. Macaques were housed in standard stainless steel primate cages (Surburban Surgical, Chicago, IL): Eight subjects were pair-housed and the other 4 were singly housed due to no compatible partners available. All animals had visual and auditory contact with other monkeys in the same room. They were fed twice daily with commercial chow (20% protein primate diet, catalog no. 2050; Harlan Teklad, Madison, WI) and also given a variety of fruit enrichment in the afternoons. Housing rooms were maintained at 18°C to 24°C (65 to 75°F), 30 to 70% humidity, and on a 12∶12 light-dark cycle (on, 6∶00 a.m.; off, 6∶00 p.m.). As a source of environmental enrichment puzzle feeders of various sorts were provided at least twice per week, destructible foraging one time per week in addition to daily fruit or yoghurt cup.

All procedures were performed by highly trained animal technicians or veterinary staff. To minimize distress and suffering during vaccination or blood withdrawal, animals were anesthetized for all procedures. Blood draws were limited to the maximum volume allowed over the duration of the experiment. After each vaccination macaques were monitored for 14 days for behavioural changes, body temperature and redness or swelling at the sight of injection. Transient inflammatory responses were observed in only 7 of 12 animals which resolved in all but 1 animal within 24 hours, with the local reaction lasting for 3 days for the additional animal. No animal required treatment for raised body temperature. A full blood count and blood chemistry was taken at each blood draw, some animals displayed slight anaemia, but values were not clinically relevant and attributed to the large blood draw required for the number of immunological assays. All animals were returned into the colony at the end of the study for use in other experiments or breeding programs (as appropriate).

### Animals and Immunizations

Female C57BL/6JOlaHsd (C57BL/6) or ICR (CD-1) mice aged at least 6 weeks (Harlan, UK) were given intramuscular (i.m.) immunizations into the musculus tibialis with a total volume of 50 µl of vaccine diluted in endotoxin-free PBS using a 29G 0.5 ml insulin syringe (BD). Each mouse experiment presented is representative of at least 2 experiments.

Rhesus macaques were screened for background ELIspot responses to TRAP and ME peptides (no response in any animal was detected) and divided into two experimental groups, based on achieving approximate equivalence of age and weight. The control (ME-TRAP) group comprised 6 animals with a median weight of 9.49 kg (range, 7.97–10.65) and median age of 4.8 years (range, 4.5–8.1); and the test (hIi-ME-TRAP) group comprised 6 animals with a median weight of 7.97 kg (range, 6.55–10.74) and a median age of 4.7 years (range 4.0–7.8). Animals received i.m. immunizations with 5×10^7^ iu ChAd63.ME-TRAP (corresponding to 2.9×10^9^ vp) or 5×10^7^ iu ChAd63.hIi-ME-TRAP (corresponding to 5.7×10^9^ vp) in a total of 0.3 ml of endotoxin-free PBS into the left deltoid muscle. At week 8 after priming, animals that had received ChAd63.ME-TRAP were boosted with 8×10^7^ plaque-forming units (PFU) MVA.ME-TRAP and animals that had received ChAd63.hIi-ME-TRAP were boosted with 8×10^7^ PFU MVA.hIi-ME-TRAP. These boost vaccinations were given into the right (contralateral) deltoid muscle. Blood samples were taken pre-vaccination, on the day of vaccination (week 0) and at 2, 4, 6, 8, 9, 11 and 20 weeks after the first vaccination.

### Antigens for In vitro Restimulation

For murine studies, cellular immune responses to TRAP were measured using *in vitro* restimulation with the synthetic peptides T9 (LNDNAIHLYVNVFSNNAKEI) and T20 (GQGINVAFNRFLVGCHPSDG), which are known to encompass H-2^b^ restricted CD4^+^ T cell and CD8^+^ T cell epitopes, respectively [28]; or with a single pool of synthetic peptides (20-mers overlapping by 10) spanning the entire TRAP sequence (Peptide Protein Research) [1]. These 57 peptides were divided into 6 pools of 10 peptides for measurement of cellular responses to TRAP in macaques. Responses to ME were measured using a single pool of 20 peptides corresponding to the individual epitopes that comprise the multi-epitope string (Peptide Protein Research) [1]. To measure the T cell response against CD74 in murine and macaque studies, 15-mer peptides overlapping by 11 (Mimotopes) and spanning the length of mIi (NCBI RefSeq NP_034675.1; isoform 2) or hIi (NCBI RefSeq NP_004346.1; isoform b) were synthesised (Table S1). For the 21 peptides from hIi that were not identical to the orthologous macaque Ii sequence (NCBI RefSeq XP_001099491.2; isoform 2), additional peptides corresponding to the cognate macaque sequences were synthesized (Mimitopes).

### ELIspots

For detection of murine antigen specific IFN-γ producing cells, mouse splenocytes pre-treated with Ammonium-chloride-potassium (ACK) lysis buffer were stimulated with the relevant peptide(s) at a final concentration of 2 µg/ml for 18–20 hours in IPVH-membrane plates (Millipore) coated with 5 µg/ml anti-mouse IFN-γ antibody AN18 (Mabtech). IFN-γ SFU were detected by staining the membranes with anti-mouse IFN-γ biotin (1 mg/ml) (R46A2) (Mabtech) followed by streptavidin-alkaline phosphatase (1 mg/ml) (Mabtech) and development with AP conjugate substrate kit (BioRad). Spots were enumerated using an ELIspot Reader System ELR04 (AID GMbH). Samples were plated in duplicate and the mean of the counts in the two wells was taken for calculation of SFU per 10^6^ cells. Background frequencies were subtracted (number of IFN-γ SFU per 10^6^ cells in the absence of peptide stimulation).

Macaque IFN-γ ELIspot assays were performed as previously described [29] using precoated ELISpot^PLUS^ kits according to the manufacturer’s recommendation (Mabtech, USA). All tests were performed in duplicate using peptide pools at a final concentration of 2 µg/ml (human Ii chain) or 5 µg/ml (ME and TRAP), with incubation for 12–18 hours at 37°C in 5% CO_2_. Enumeration and calculations were performed as above. A response was considered positive if the SFU exceeded mean background plus two standard deviations (SD) and was >50 SFU per 10^6^ cells.

### Intracellular Cytokine Staining (ICS)

Mouse splenocytes or PBMCs were treated with ACK to lyse erythrocytes prior to stimulation at 37°C for 6 hours with 2 µg/ml of TRAP peptide pool or hIi peptide pool with 1 µg/ml Golgi-Plug (BD). Following surface labelling with anti-CD4-e450 and anti-CD8-PerCPCy5.5 (all eBioscience) and staining with fixable Live/Dead Aqua (Invitrogen), cells were fixed with neutral buffered formalin solution containing 4% formaldehyde (Sigma) for 5 minutes at 4°C, prior to intracellular staining with anti-TNF-α Alexa488, anti-IL-2-PE and anti-IFN-γ Alexa647 (eBioscience) antibodies diluted in Perm-Wash buffer (BD).

ICS of macaque PBMCs was performed as previously described [30]. Briefly, cells were incubated overnight with a single pool of TRAP peptides at 2 µg/ml final concentration, anti-CD28 (clone L293; BD Biosciences), anti-CD49d (clone 9G; BD), and CD107a PE (clone H4A3; BD) antibodies and 5 µg per test of Brefeldin A and Golgi Stop. For surface staining, cells were labelled with anti-CD3 v500 (clone SP34; BD Biosciences), anti-CD8 Pacific Blue (clone RPA-T8; BD Biosciences), anti-CD4 PerCPCy5.5 (clone L200; BD Biosciences), anti-CD14 PE-Texas Red (clone RMO52; Beckman Coulter), and anti-CD19 PE-Texas Red (clone JA.119; Beckman Coulter), washed twice with FACS buffer, and fixed with 1% paraformaldehyde. Cells were then permeabilized with 0.1% Saponin buffer, intracellularly stained with anti-IFN-γ FITC (clone 4S.B3; BD Biosciences), anti-TNF-α Alexa700 (clone MAb11; BD Biosciences), anti-IL-2 APC (clone MQ1-17 H12; BD Biosciences). After 30 minutes incubation, cells were washed twice with Saponin buffer, and fixed with 1% paraformaldehyde.

For the analysis of memory response, frozen PBMC samples were thawed and rested for 6 hours at 37°C in media containing 10 U/ml of Benzonase (Novagen). Cells were then stimulated for a total of 16 hours with a single pool of TRAP peptides at 2 µg/ml final concentration, anti-CD28 APC (clone CD28.2, eBioscience), anti-CD49d (clone 9F10, eBioscience), Golgi-Stop and Golgi-Plug (BD Bioscience). Surface staining with CD95-bi (clone DX2, eBioscience) av-qDot565 (Invitrogen), CD4-APC H7 (clone L200, BD Bioscience), CD14 PE-Cy7 (clone M5E2, BioLegend), CD20 PE-Cy7 (clone 2H7, BioLegend) and live-dead Aqua was followed by fixation in 10% neutral buffered formalin (Sigma), prior to intracellular staining for CD3-PE (clone SP34-2, BD Bioscience), CD8 APC-H7 (clone RPA-T8, BioLegend) and IFN-γ FITC (clone 4S.B3, BioLegend) in Perm-Wash Buffer (BD).

All flow cytomery data was analysed using FlowJo (TreeStar) with further analysis of polyfunctionality performed through the use of Pestle v1.7 and SPICE v5.2 [31]. Typically, murine antigen specific cells were identified by gating based on live cells, size, doublet negative and either CD4^+^CD8^−^ or CD4^−^CD8^+^ (Fig. S1A) while macaque cells were gated based on size, doublet negative, CD3^+^, and either CD4^+^CD8^−^ or CD4^−^CD8^+^ (Fig. S1B). Statistical analysis was performed using Prism v5.0 c (Graphpad).

### Antibody Responses

Antibody response to TRAP, human Ii chain and macaque Ii chain were measured using a luciferase immunoprecipitation system (LIPS) as previously described [32]. The assay is based on binding of immobilised antibodies to a fusion protein of TRAP (*P. falciparum* 3D7 sequence) (human Ii or macaque Ii) and *Renilla* luciferase (rLuc). Briefly, serum samples were incubated for 1 hour with a cell lysate from 293 cells transfected with a TRAP-rLuc (hCD74-rLuc or macCD74-rLuc) expression plasmid, prior to incubation with Protein A/G UltraLink Resin beads (ThermoScientific) in MultiScreen HTS membrane Barex plates (Millipore) for 1 hour. Unbound lysate and antibodies were removed by washing the plates prior to quantification of bound rLuc activity using *Renilla* luciferase assay system (Promega) and a Varioskan Flash luminometer (Thermo). Antibody levels are expressed as log_10_ luminescence units.

## Results

### Enhanced Immunogenicity of ChAd63.mIi-ME-TRAP in Mice

**Figure 1:**
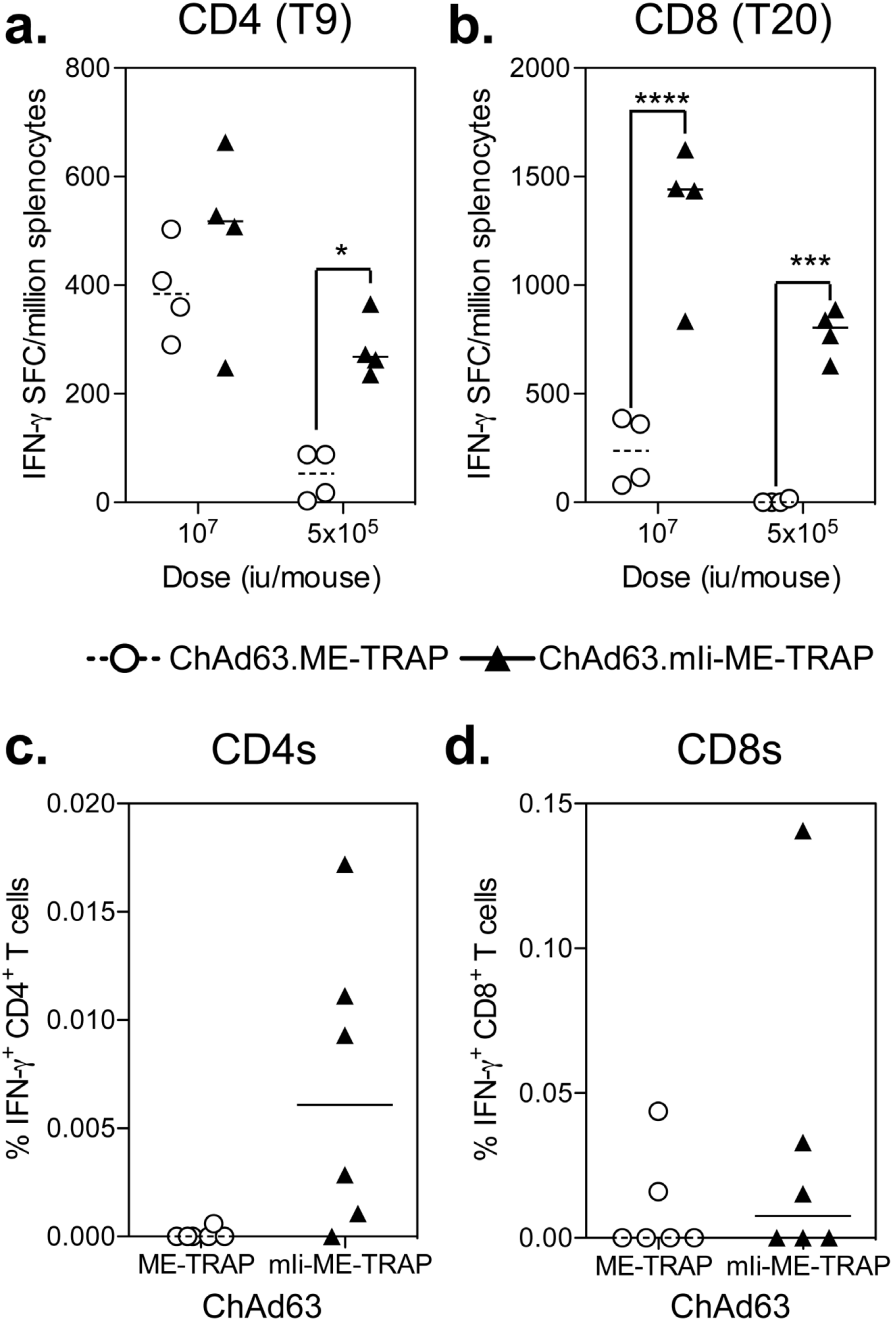
Effect of fusion of mIi to ME-TRAP in mice immunized with ChAd63 vectors. a. & b.) C57BL/6 mice were vaccinated i.m. with 10^7^ or 5×10^5^ iu of ChAd63.ME-TRAP (circles) or ChAd63.mIi-ME-TRAP (triangles) and spleens were harvested two weeks later to measure the response to the dominant CD4^+^ (a.) and CD8^+^ (b.) T cell epitopes by IFN-γ ELISpot. c. & d.) Outbred CD-1 (ICR) mice were vaccinated i.m. with 10^7^ iu of ChAd63.ME-TRAP (circles) or ChAd63.mIi-ME-TRAP (triangles) and spleens harvested two weeks later to measure IFN-γ producing TRAP specific CD4^+^ (c.) and CD8^+^ (d.) T cell responses by ICS. Points represent individual mice after subtraction of background responses and lines represent the median. ****p<0.0001, ***p<0.01, *p<0.05 compared to ChAd63.ME-TRAP (2-way ANOVA with Bonferroni post-test).

To determine whether fusion of ME-TRAP to murine invariant chain (mIi) could enhance the antigen specific T cell response following vaccination with ChAd63 vectors, either inbred (C57BL/6) or outbred (CD-1) mice were vaccinated i.m. with ChAd63.ME-TRAP or ChAd63.mIi-ME-TRAP, and the cellular immune responses against TRAP were analysed two weeks later, corresponding to the peak in immunogenicity [28], by IFN-γ ELIspot or IFN-γ ICS. In C57BL/6 mice, statistically significantly (p<0.05) higher frequencies of CD4^+^ T cells responding to T9 peptide were observed with ChAd63.mIi-ME-TRAP vaccination compared with ChAd63.ME-TRAP at a vector dose of 5×10^5^ iu (Fig. 1a), and a non-significant trend in the same direction was observed at a dose of 10^7^ iu. Significantly (p<0.001) higher frequencies of CD8^+^ T cells responding to T20 peptide were observed at both these doses (Fig. 1b). Due to the increased heterogeneity and consequently decreased level of immunogenicity often observed in outbred mice [33], we chose to increase the dose of ChAd63 and immunogenicity was measured by ICS to the total pool of TRAP peptides. Two weeks after vaccination of CD-1 mice a statistically significant difference in the frequency of CD4^+^ and CD8^+^ T cell responses were not detected, but 5/6 and 3/6 mice vaccinated with ChAd63.hIi-ME-TRAP had detectable CD4^+^ and CD8^+^ T cell responses, respectively, compared with 1/6 and 2/6 mice, respectively, in the control group (ChAd63.ME-TRAP).

### Head-to-head Comparison of ChAd63.hIi-ME-TRAP and ChAd63.mIi-ME-TRAP in Mice

**Figure 2:**
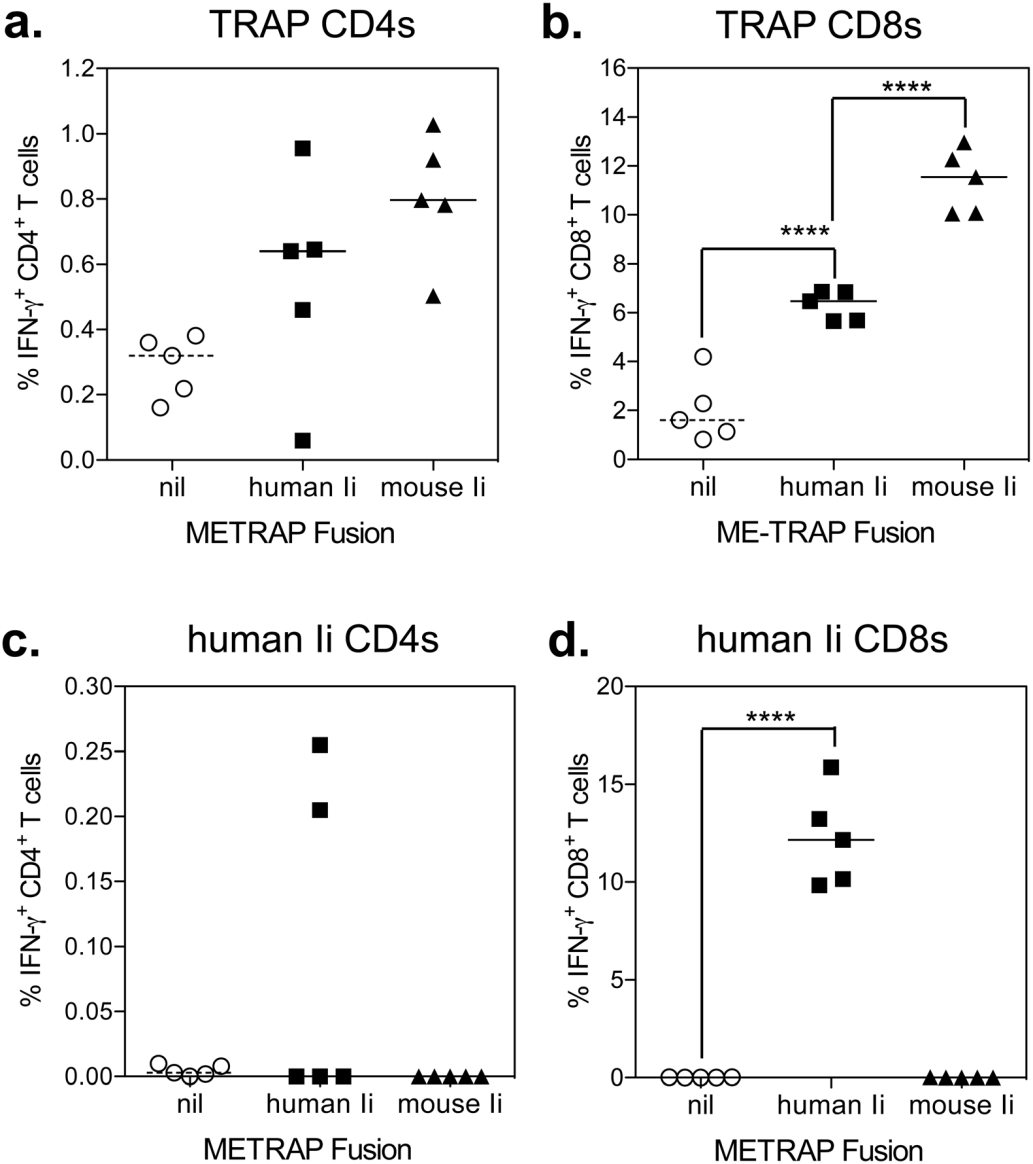
Effect of hIi or mIi fusion to ME-TRAP in mice immunized with ChAd63 vectors. C57BL/6 mice were vaccinated i.m. with 10^8^ iu of ChAd63.ME-TRAP (circles), ChAd63.hIi-ME-TRAP (squares) or ChAd63.mIi-ME-TRAP (triangles) with spleens and sera harvested two weeks after vaccination. T cell responses to TRAP (a. & b.) or hIi (c. & d.) peptides were measured by ICS. Points represent individual mice after subtraction of background responses and lines represent the median. ****p<0.0001 compared to ChAd63.ME-TRAP (2-way ANOVA with Bonferroni post-test).

We next determined whether the human ortholog of the invariant chain (hIi) could also enhance murine T cell responses to the same degree as mIi when fused to the same antigen. C57BL/6 mice were immunized with 10^8^ iu of ChAd63.ME-TRAP, ChAd63.hIi-ME-TRAP or ChAd63.mIi-ME-TRAP, with spleens and sera harvested two weeks post-vaccination. Consistent with the previous experiments, statistically significantly higher frequencies of CD4^+^ and CD8^+^ T cells were again observed in C57BL/6 mice vaccinated with ChAd63.mIi-ME-TRAP compared with the control vector (ChAd63.ME-TRAP). A statistically significant enhancement, although less pronounced, was also observed in animals vaccinated with ChAd63.hIi-ME-TRAP (Fig. 2a and 2b).

To determine whether the apparently smaller enhancement in cellular immune responses observed using hIi in mice could have been associated with induction of competitive responses against hIi itself, T cell responses against hIi were enumerated. Two mice demonstrated detectable CD4^+^ T cell responses to hIi (Fig. 2c), but all mice exhibited robust CD8^+^ T cell responses to hIi (Fig. 2d), which were of higher frequency than the anti-TRAP CD8^+^ T cell responses. In a similar experiment, cellular immune responses against mIi were assessed. As previously observed, higher CD8^+^ T cell responses were observed in mice immunised with ChAd63.mIi-ME-TRAP compared with ChAd63.ME-TRAP (Fig. S2a); while CD4^+^ and CD8^+^ T responses to murine Ii peptides were at the limit of detection, responses were similar between the two groups of mice (Fig. S2b).

### Enhanced Immunogenicity of ChAd63. hIi-ME-TRAP and MVA.hIi-ME-TRAP in Mice

**Figure 3:**
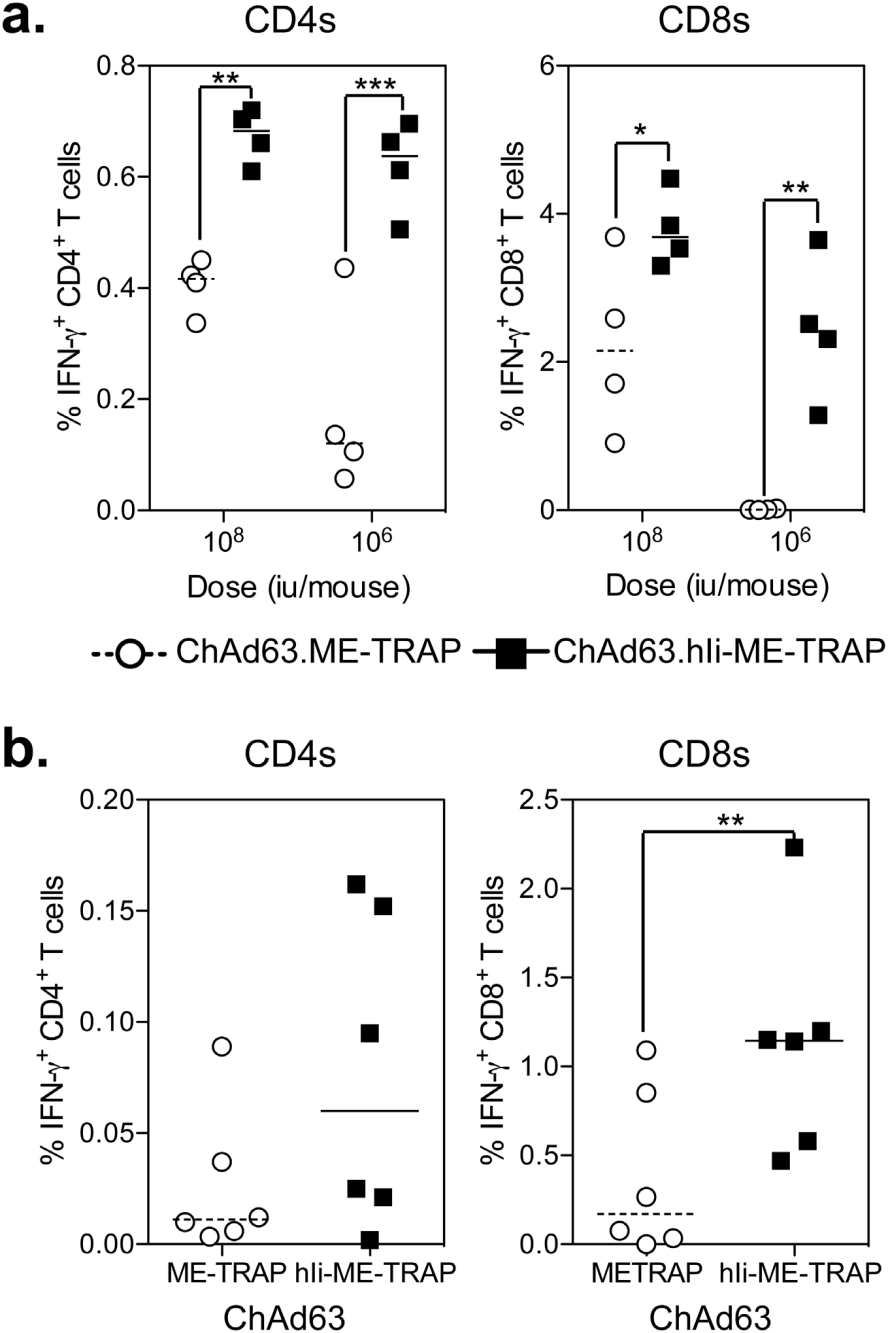
Human invariant chain fusion in mice. a.) C57BL/6 mice were immunized i.m. with 10^8^ and 10^6^ iu of ChAd63.ME-TRAP (circles) or ChAd63.hIi-ME-TRAP (squares) with spleens harvested two weeks later to measure TRAP specific CD4^+^ (left) and CD8^+^ (right) T cell responses by ICS. b.) CD-1 (ICR) mice were vaccinated i.m. with 10^8^ iu of ChAd63.ME-TRAP (circles) or ChAd63.hIi-ME-TRAP (squares) with spleens harvested 2 weeks after vaccination to measure TRAP specific CD4^+^ (left) and CD8^+^ (right) T cell response by ICS. In each graph, individual mice are denoted by a single point after background subtraction (unstimulated) and lines represent the median response per group. Data in each experiment was analysed with a two-way analysis of variance and where a significant effect of the human Ii chain was observed, a post-hoc Bonferoni test was performed, asterisks denote the level of statistical significance when compared to the control ME-TRAP vaccinated group (*p<0.05, **p<0.01, ***p<0.001).

To more thoroughly characterize the immunopotentiating capacity of hIi in mice, C57BL/6 mice were vaccinated with 10^8^ or 10^6^ iu of ChAd63.ME-TRAP or ChAd63.hIi-ME-TRAP and responses to TRAP were measured in the spleens by ICS. At both doses, a statistically significantly higher frequency of IFN-γ-producing TRAP-specific CD4^+^ and CD8^+^ T cells were observed (Fig. 3a), consistent with the previous experiment. Since the majority of CD-1 mice did not induce a response when vaccinated with 10^7^ iu ChAd63.ME-TRAP (Fig. 1), CD-1 mice were vaccinated with 10^8^ iu of ChAd63.ME-TRAP or ChAd63.hIi-ME-TRAP to determine whether human Ii chain could augment the response in an outbred population of mice. Two weeks after vaccination, a significant increase (p<0.01) in IFN-γ producing TRAP-specific CD8^+^ T cells was observed in the spleen of ChAd63.hIi-ME-TRAP vaccinated mice (Fig. 3b), with a small increase in the median response of CD4^+^ T cells (albeit not statistically significant).

**Figure 4:**
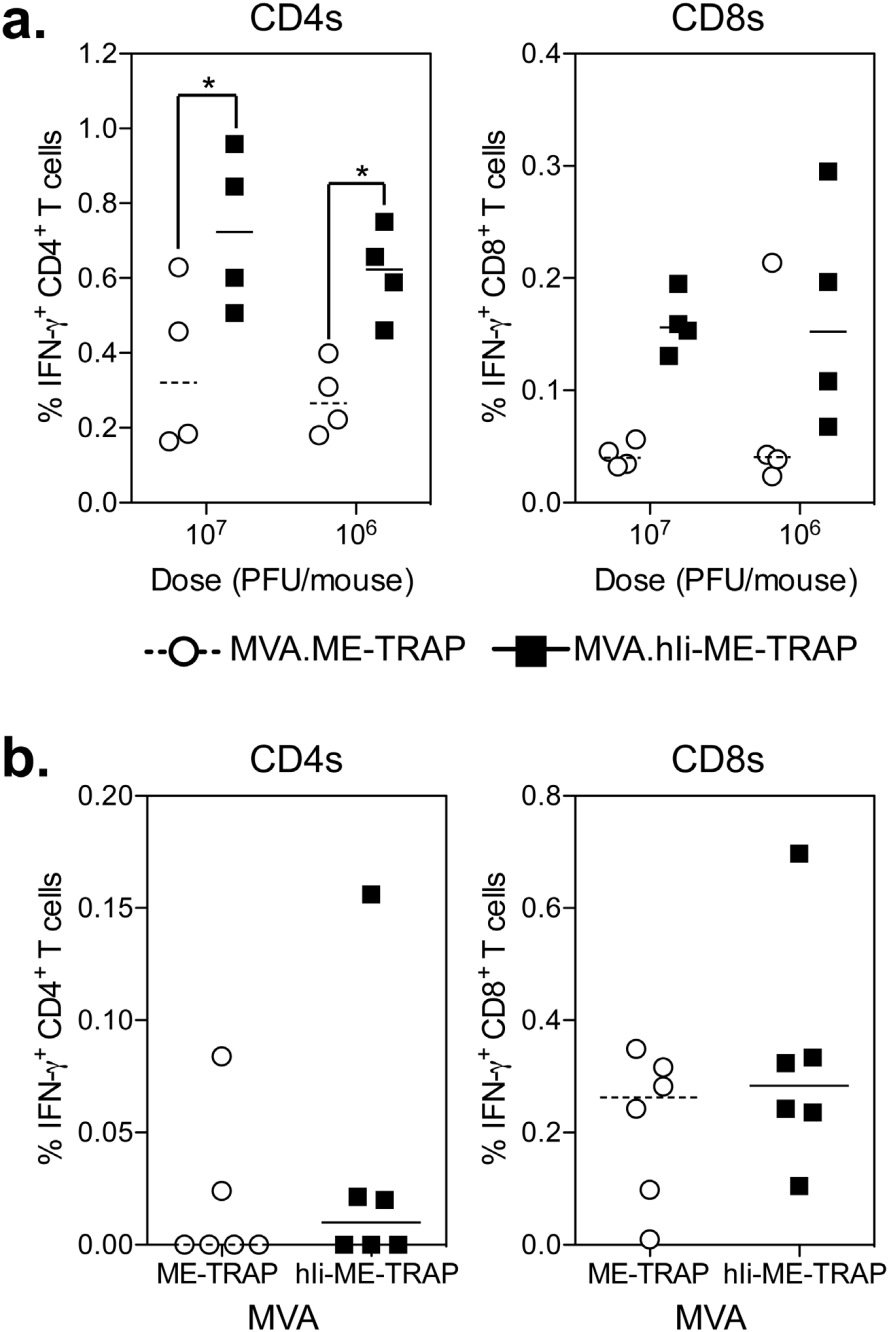
Human invariant chain in an MVA vector. a.) C57BL/6 mice were immunized i.m. with 10^7^ and 10^6^ PFU of MVA.ME-TRAP (circles) or MVA.hIi-ME-TRAP (squares) with spleens harvested 1 week later to measure TRAP specific CD4^+^ (left) and CD8^+^ (right) T cell responses by ICS. b.) CD-1 (ICR) mice were vaccinated i.m. with 10^7^ PFU of MVA.ME-TRAP (circles) or MVA.hIi-ME-TRAP (squares) and spleens harvested 1 week later to measure TRAP specific CD4^+^ (left) and CD8^+^ T cell response by ICS. In each graph, individual mice are denoted by a single point after background subtraction (unstimulated) and lines represent the median response per group. Data in each experiment was analysed with a two-way analysis of variance and where a significant effect of the human Ii chain was observed, a post-hoc Bonferoni test was performed, asterisks denote the level of statistical significance when compared to the control ME-TRAP vaccinated group (*p<0.05).

To determine the capacity of hIi fusion to increase the response when delivered using an MVA vector, C57BL/6 and CD-1 mice were vaccinated with varying doses of MVA.ME-TRAP or MVA.hIi-ME-TRAP vectors and the TRAP-specific responses in the spleen were measured by ICS 1 week post-vaccination, corresponding to the peak in immunogenicity. Consistent with previous reports the cellular immune responses induced by MVA vectors were of lower frequency than by ChAd63 vectors [28], however a statistically significant difference in the frequency of TRAP-specific CD4^+^ T cells was observed in C57BL/6 mice vaccinated with either 10^7^ or 10^6^ PFU of MVA.hIi-ME-TRAP compared with MVA.ME-TRAP (Fig. 4a). A small difference in the TRAP-specific CD8^+^ T cell response was observed in mice vaccinated with either 10^7^ or 10^6^ PFU of MVA.hIi-ME-TRAP, but was not statistically significant. In addition, no differences in the frequency of TRAP-specific IFN-γ^+^ CD4^+^ or CD8^+^ T cell responses were observed in CD-1 mice vaccinated with 10^7^ PFU MVA vectors (Fig. 4b).

In Summary, Fusion of ME-TRAP to Hii Resulted in Induction of Higher Frequencies of TRAP Specific T Cells, When Delivered Using Chad63 and MVA Vectored Vaccines, with the Greatest Enhancement Observed in Inbred Mice Vaccinated with Chad63 Vectors.

### Enhanced Immunogenicity of hIi-ME-TRAP in Mice in a Heterologous Prime-boost Regimen

**Figure 5:**
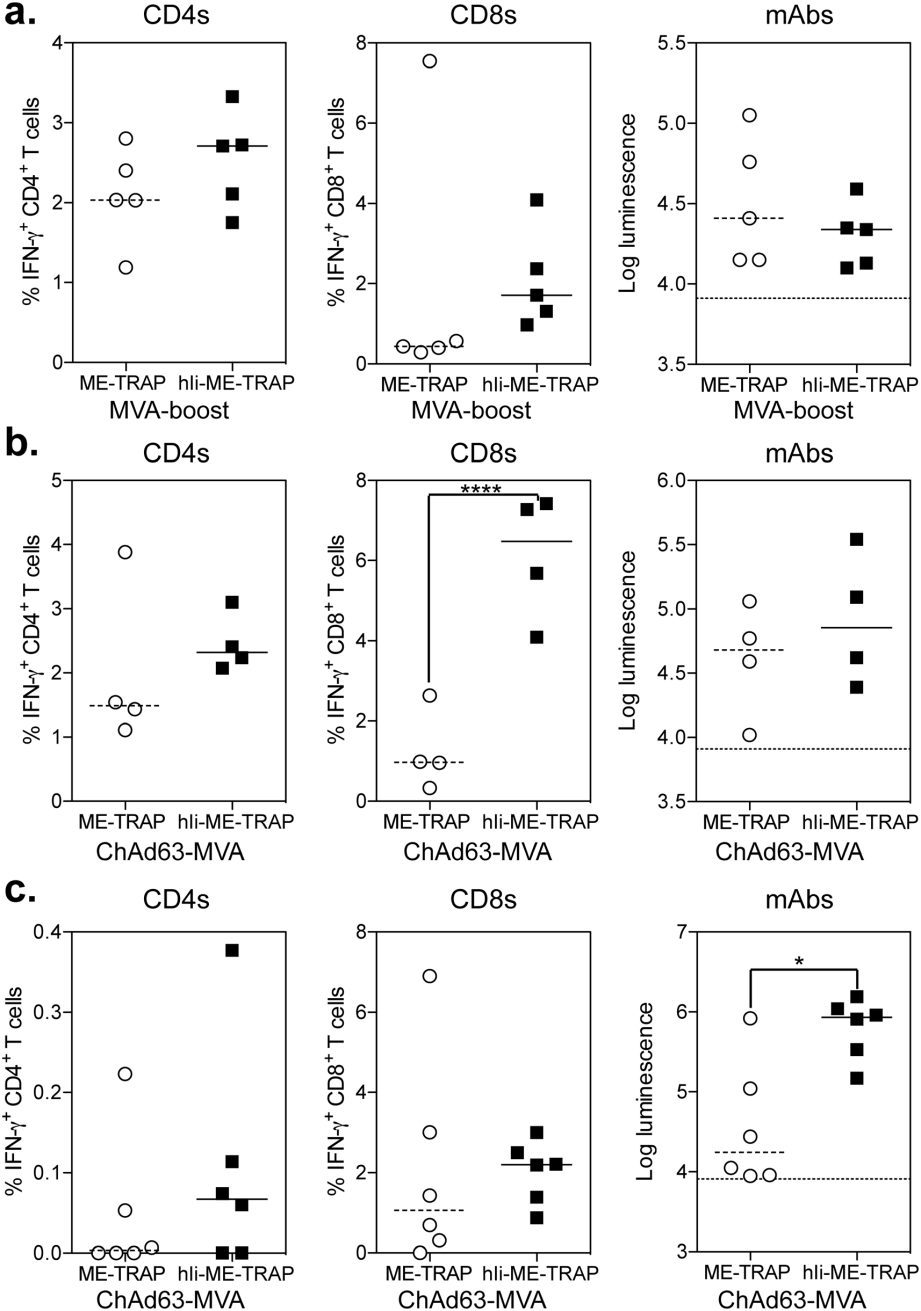
ChAd63 MVA prime-boost regimens in mice. a.) C57BL/6 mice were immunized i.m. with 10^6^ iu of ChAd63.ME-TRAP and 8 weeks later boosted with either 10^6^ PFU of MVA.ME-TRAP (circles) or MVA.hIi-ME-TRAP (squares). b.) C57BL/6 mice were immunized i.m. with 10^6^ iu of ChAd63.ME-TRAP (circles) or ChAd63.hIi-ME-TRAP (squares) and boosted 8 weeks later with the 10^6^ PFU of MVA vector containing the same insert. c.) CD-1 (ICR) mice were immunized i.m. with 10^8^ iu of ChAd63.ME-TRAP (circles) or ChAd63.hIi-ME-TRAP (squares) and boosted 8 weeks later with the 10^6^ PFU of MVA vector containing the same insert. In all experiments, spleens and sera were harvested 1 week after the MVA vaccination, individual mice are denoted with a single point and lines represent the median response per group. TRAP specific CD4^+^ (left) and CD8^+^ (middle) T cells responses were measured by ICS with the data from each experiment analysed with a 2-way analysis of variance and a post-hoc Bonferoni positive test; asterisk denotes the level of statistical significance compared to the control ME-TRAP vaccinated group (***p<0.001). Abs against TRAP were measured by LIPS assay (right), the dotted line in each graph denotes the luminescence of a naïve mouse. Data from each experiment was analysed with a Mann-Whitney test, a significant effect of hIi was only observed in outbred mice (*p = 0.0152).

To assess the immunopotentiating capacity of hIi in a clinically-relevant heterologous prime-boost vaccination regimen, comprising ChAd63 prime and MVA boost [6], [9], we first assessed whether MVA.hIi-ME-TRAP could act as a more potent boost than MVA.ME-TRAP following an identical prime vaccination. Therefore C57BL/6 mice were vaccinated with 10^6^ iu of ChAd63.ME-TRAP and 8 weeks later received a boost vaccination with 10^6^ PFU of either MVA.ME-TRAP or MVA.hIi-ME-TRAP. The small differences in CD4^+^ and CD8^+^ T cell responses between the two groups of mice (Fig. 5a) were not statistically significant. There was also no difference in the level of TRAP-specific antibodies detected by the LIPS assay.

We next determined the effect of using hIi-fused antigen in both the priming ChAd63 and boosting MVA vaccinations. C57BL/6 mice were immunised with 10^6^ iu of ChAd63.ME-TRAP or ChAd63.hIi-ME-TRAP, followed 8 weeks later by 10^6^ PFU of MVA vector expressing the cognate antigen. In mice immunised with ChAd63.hIi-ME-TRAP and MVA.hIi-ME-TRAP a dramatically higher CD8^+^ T cell response was observed after the MVA boost (Fig. 5b), compared with mice immunised with the unmodified vectors in the same regimen. A small but not statistically significant difference in CD4^+^ T cell responses was observed, while there was no difference in antibody responses.

A number of studies have suggested that quality of the immune response can impact the overall protective capacity [34], [35]. However protection from liver-stage malaria is more strongly correlated with the overall frequency of IFN-γ producing cells [9], [36]. To ensure that fusion to the Ii chain was not skewing the response we also investigated the polyfunctionality of TRAP specific responses, in terms of the production of IFN-γ, TNF-α and IL-2. Fusion of ME-TRAP to hIi did not alter the proportion of either CD4^+^ of CD8^+^ T cells secreting any combination of these three cytokines, but rather induced higher frequencies of cells, regardless of cytokine expression profile (Fig. S3a).

We subsequently tested the ChAd63-MVA regimen in CD-1 mice, using a priming dose of 10^8^ iu of ChAd63 and the same dose of the MVA boost (10^6^ pfu). Higher frequencies of antigen-specific CD4^+^ and CD8^+^ T cell responses (albeit not statistically significant) and higher antibody responses (p<0.05) were observed in mice given prime-boost vaccinations with ChAd63 and MVA expressing hIi-ME-TRAP compared with ME-TRAP (Fig. 5c).

In summary, vaccination with a ChAd63-MVA prime-boost regimen in which both vectors expressed hIi-ME-TRAP leads to an overall increase in the immune responses compared to the same regimen in which both vectors expressed ME-TRAP.

### Enhanced Immunogenicity of hIi-ME-TRAP in Non-human Primates

**Figure 6:**
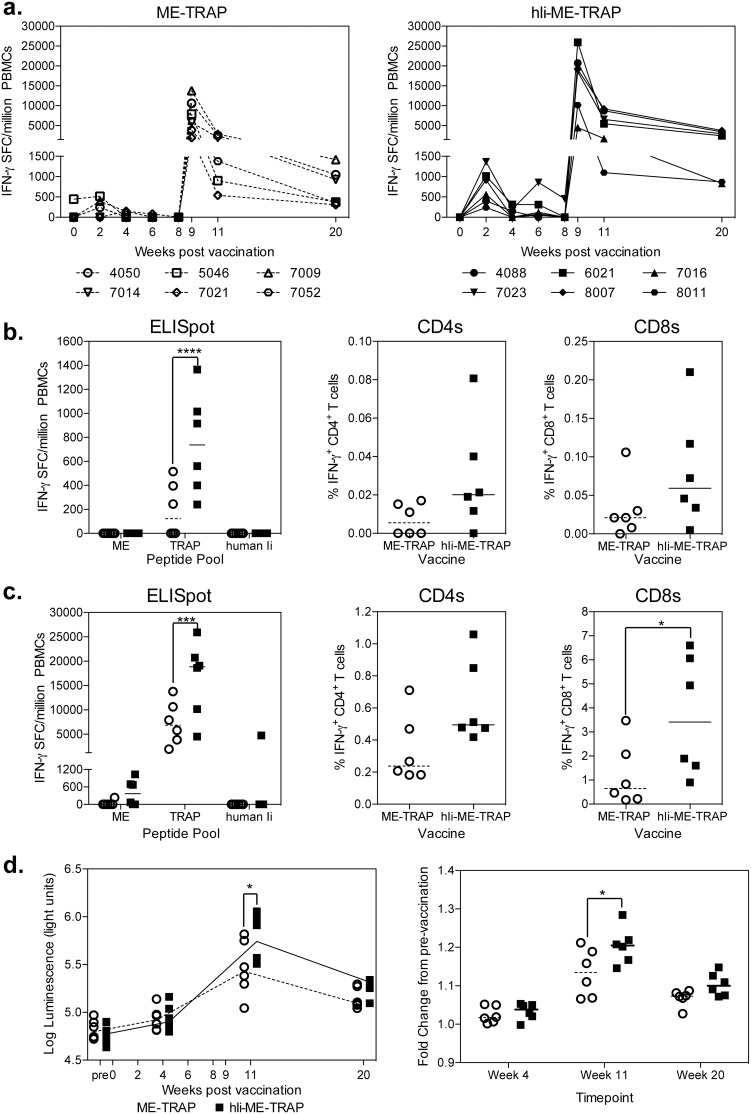
ChAd63-MVA prime boost regimen in non-human primates. Male rhesus macaque were vaccinated i.m. with either ChAd63.ME-TRAP followed 8 weeks later by MVA.ME-TRAP (open symbols), or ChAd63.hIi-ME-TRAP followed 8 weeks later by MVA.hIi-ME-TRAP (closed symbols). Blood samples were taken on the day of vaccination, weeks 2, 4, 6, 8 following ChAd63 vaccination and 1 (9), 3 (11) and 12 (20) weeks following MVA vaccination. a.) The graphs represent the summed response to TRAP peptides over the course of the experiment for animals immunised with ME-TRAP (left) or hIi-ME-TRAP (right) vectors. The data was analysed with a 2-way anova (repeated measures) demonstrating a significant effect of the human Ii chain (p = 0.0182) and a post-hoc Bonferoni positive test to compare the two groups at each timepoint. b.) Graphs represent the response at week 2 to ME, TRAP and human Ii chain peptides measured by ELISpot at (left) and the IFN-γ TRAP specific response of CD4^+^ (centre) or CD8^+^ (right) T cells as measured by ICS at this same time. Each data set (ELISpot or ICS) was analysed with a 2-way analysis of variance and where a significant effect of human Ii was observed (ELISpot p = 0.0080), a post-hoc Bonferoni positive was performed (asterisks denote the level of significance) (****p<0.0001). c.) Graphs represent the response at week 9 to ME, TRAP and human Ii chain peptides measured by ELISpot at (left) and the IFN-γ TRAP specific response of CD4^+^ (centre) or CD8^+^ (right) T cells as measured by ICS at this same time. Each data set (ELISpot or ICS) was analysed with a 2-way analysis of variance and where a significant effect of human Ii was observed (ELISpot p = 0.0094, ICS p = 0.0274), a post-hoc Bonferoni positive was performed (asterisks denote the level of significance) (***p<0.01, *p<0.05). d.) Graphs represent the antibody response to TRAP over the course of the vaccination (left panel) or as a fold increase from the pre-vaccination timepoint. Data was analysed with a 2-way analysis of variance (repeated measure) with a significant effect of the human Ii chain observed (timecourse p = 0.0443, fold change p = 0.0341) with a post-hoc Bonferoni positive test to determine the significance at each timepoint, asterisks denote the level of significance (*p<0.05).

To assess the ability of hIi fusion to augment immune responses following vectored vaccination of non-human primates, two groups of male rhesus macaques were given prime-boost vaccinations with ChAd63.ME-TRAP followed by MVA.ME-TRAP or ChAd63.hIi-ME-TRAP followed by MVA.hIi-ME-TRAP. TRAP specific cellular immune responses were measured by IFN-γ ELISpot and ICS. Two weeks after ChAd63 vaccination, a TRAP-specific ELIspot response was evident in all 6 animals vaccinated with hIi-ME-TRAP (Fig. 6b) while 3 of the 6 animals vaccinated with ME-TRAP demonstrated a detectable TRAP-specific response. The median ELISpot response to TRAP in ChAd63.hIi-ME-TRAP vaccinated macaques was higher than in the ChAd63.ME-TRAP group (p<0.0001). At this time, no responses to either the ME peptide pool or human Ii chain peptides were detected. No statistically significant difference between the two groups was observed when CD4^+^ and CD8^+^ T cell responses were enumerated by ICS at the same timepoint, though there was a trend in the same direction as the ELISpot results (Fig. 6b centre and right). TRAP-specific ELIspot responses declined after week 2 until after the MVA boost at week 8 (Fig. 6a). One week post-MVA boost (week 9) a strong TRAP specific response was observed in all vaccinated rhesus macaques (Fig. 6a), with a statistically significantly higher median TRAP response observed in animals vaccinated with hIi-ME-TRAP-expressing vectors compared with ME-TRAP (Fig. 6a, c). At this time-point, 1 of 6 animals in the ME-TRAP group had a detectable response to ME peptides (235 SFC/million PBMCs) compared to four of the six animals in the hIi-ME-TRAP group (median response in responding animals, 682 SFC/million PBMCs) (Fig. 6a left). A detectable reponse to hIi peptides was observed in one macaque, which also exhibited the highest TRAP-specific ELIspot response. This response was directed against peptides from hIi which do not share sequence identity with the macaque Ii ortholog (Fig. S4a, b). Fine mapping of this response on frozen PBMCs cells, demonstrated a response to a single region corresponding to two changes in the amino-acid sequence at position 85–87 (SQN to TQS) (Data not shown). No antibody response against either hIi or macaque Ii was observed in this macaque (Fig. S4c) or any other vaccinated macaque (Fig. S4d) as measured by the LIPs assay.

ICS analysis performed at week 9 confirmed the higher TRAP specific cellular responses in animals vaccinated with hIi-ME-TRAP-expressing vectors, with a statistically significant increase in CD8^+^ T cells observed (Fig. 6c right). The cellular phenotype of the response was also assessed in terms of the ability of cells to upregulate CD107a surface expression (a marker of degranulation) in addition to IFN-γ, TNF-α and IL-2 production upon restimulation with TRAP peptide pool. Consistent with the data from the murine studies, vaccination with hIi-ME-TRAP did not alter the proportions of CD4^+^ (Fig. S3b top panel) or CD8^+^ (Fig. S3b bottom panel) T cells able to simultaneously produce any combination of cytokine analysed and upregulate CD107a. Higher frequencies of antigen-specific T cells were observed for most of the cellular phenotypes assessed (Fig. S3b graphs), indicating that the functionality of the response was not substantially altered by vaccination with vectors encoding hIi-fused antigen versus unmodified antigen.

Antibodies to TRAP were also analysed following prime-boost vaccination with ChAd63-MVA. In both groups of animals, increases in antibody levels were observed after vaccination compared to pre-vaccination levels, with the highest responses observed at the week 11 timepoint (3 weeks after MVA boost) (Fig. 6d). At week 11, fusion of ME-TRAP to hIi in the vectors was associated with statistically significantly higher antibody response (Fig. 6d left panel) and higher increases in antibody levels compared with the pre-vaccination timepoint (Fig. 6d right panel).

**Figure 7:**
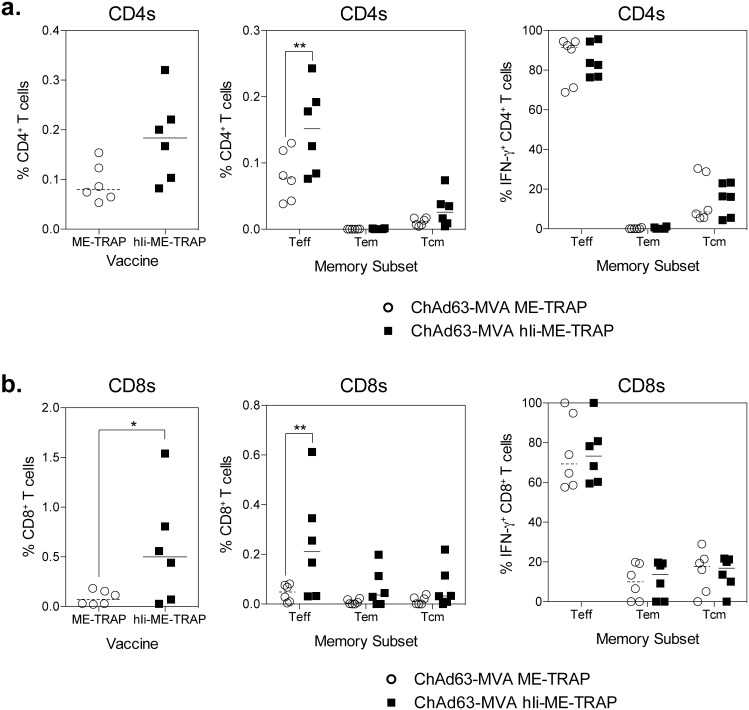
TRAP specific response in non-human primates 12 weeks after MVA boost. Frozen PBMCs samples taken from male rhesus macaque 12 weeks after the MVA boost (Week 20 sample) were thawed and restimulated for 16 hours with TRAP peptides prior staining for IFN-γ production and memory/effector T cell markers. a.) Graphs represent the frequency of IFN-γ^+^ CD4^+^ T cells (left panel), frequency of T cell subset^+^ CD4^+^ T cells (central panel) or the frequency of each T cell subsets as a percentage of IFN-γ^+^ CD4^+^ T cells (right panel). b.) Graphs represent the frequency of IFN-γ^+^ CD8^+^ T cells (left panel), frequency of T cell subset^+^ CD8^+^ T cells (central panel) or the frequency of each T cell subsets as a percentage of IFN-γ^+^ CD8^+^ T cells (right panel). Analysis with a two-way anova showed a significant effect of hIi on the frequency of IFN-γ^+^ T cells (p = 0.0227), frequency of CD4^+^ T cells subset (p = 0.0117) and CD8^+^ T cell subsets (p = 0.0057) T cells; post-hoc Bonferoni positive test were performed to determine the effect the hIi on either CD4 or CD8 cells, or each T cell subset, asterisk denote the level of significance (*p<0.05, **p<0.01).

To determine whether vaccination with hIi-ME-TRAP lead to an enhanced maintenance of the response, frozen PBMCs from week 20 (11 weeks after MVA boost) were stimulated overnight with TRAP peptides and analysed for the expression of memory markers CD95 and CD28 [37]–[40]. Consistent with the fresh IFN-γ ELISpot data, higher TRAP specific cells IFN-γ^+^ responses were observed in the group of animals vaccinated with hIi-ME-TRAP fusion (Figure 7). When IFN-γ^+^ TRAP specific cells were subdivided into CD28^+^ CD95^−^ T effector cells (Teff), CD28^+^ CD95^−^ T effector memory cells (Tem) or CD28^+^ CD95^+^ T central memory cells (Tcm) a statistically significant increase in Teff CD4^+^ (Figure 7A left central) and Teff CD8^+^ T cells (Figure 7B central panel) were observed in hIi-ME-TRAP vaccinated animals. Importantly vaccination with hIi-ME-TRAP did not alter the proportion of cells in each T cell subset (Figure 7 right panels), but led to an overall increase in the response 11 weeks after MVA boost.

In summary, ChAd63-MVA prime-boost vaccination with vectors encoding ME-TRAP fused to hIi resulted in augmented immunogenicity in non-human primates compared with unfused antigen, both in terms of T cell responses and anti-TRAP immunoglobulin, with increased T cells responses maintained up to 11 weeks after MVA boost.

## Discussion

Despite large efforts to reduce malaria transmission through the wide-spread deployment of insecticides and bed nets, malaria continues to kill around 0.6 million per annum, mostly in sub-Saharan Africa and primarily in children under 5 [41]. Due to the absence of symptoms during the pre-erythrocytic stage of malaria, a vaccine which eliminates the infection at this stage is highly desirable. Studies in the 1960s demonstrated the ability of irradiated sporozoites to induce sterile protection and since this time both antibodies and CD8 T cells have been shown to play a significant role [42].

The leading malaria vaccine, RTS,S/AS01 which achieves up to 50% efficacy, appears to work primarily through the induction of high levels of antibodies against the circumsporozoite protein (CS) to block infection of hepatocytes [43]. Alternatively, vaccination with viral vectors aims to induce CD8^+^ T cells capable of killing infected hepatocytes [7]. The most clinically advanced approach is heterologous vaccination with ChAd63 and MVA vectors expressing ME-TRAP which achieves the second highest level efficacy to date, and protection is correlated to the level of CD8^+^ T cells [9]. To improve the levels of efficacy with this regimen higher T cell responses may be required.

Ii is a type II integral membrane protein which forms a trimer, associates with MHC Class II molecules in the endoplasmic reticulum and mediates trafficking into the endosomal-lysosomal pathway [44]. Due to its role in MHC class II presentation, fusion of Ii to a recombinant antigen of interest was originally investigated as a mechanism to increase CD4^+^ T cell responses [12]–[15]. Following several reports demonstrating an increase in CD8^+^ T cell immunogenicity [16]–[20], in this study we have assessed the ability of fusion of ME-TRAP to Ii chain as a means of increasing T cell responses induced by vaccination with chimpanzee adenovirus and MVA vectors.

Initial experiments in mice indicated that both mouse and human Ii could increase CD8^+^ T cell responses in inbred and outbred strains of mice. Immunopotentiation by the human and murine Ii fusions demonstrated a degree of species specificity in mice with a greater relative increase in CD8^+^ T cell responses observed with the murine Ii fusion (Fig. 2). However T cell responses to the human but not the murine sequence were observed, suggesting that the reduced ability of the human sequence to augment TRAP specific responses could be a result of a competing T cell response. Given the reduced effectiveness of human Ii chain, and T cell reactivity against it in mice, we hypothesised that a similar reactivity against mouse Ii chain would be observed in humans and therefore we chose to progress testing ME-TRAP fused to human Ii in non-human primates.

Following the protective and most immunogenic clinical regimen, ChAd63 followed 8 weeks later by an MVA boost, we observed a significantly higher immune response in macaques vaccinated with hIi-ME-TRAP compared with the unmodified antigen construct. An augmentation in both CD4^+^ and CD8^+^ T cell responses was seen after single administration of ChAd63, while the most pronounced difference was observed after MVA boost (Fig. 5). A significant improvement in antibodies was also observed after MVA boost which we believe is the first evidence of an Ii fusion increasing antibodies induced by vectored vaccine immunisation. Detection of the enhanced humoral immunogenicity mediated by fusion to Ii may be aided by the vaccine regimen, which is known to induce high titre antibodies relative to administration of protein in adjuvant [45], [46]. Importantly an increase in IFN-γ producing T cells did not arise at a cost of other cytokine producing populations and no change in the polyfunctionality of the T cell response was observed in either mice or macaques based on analysis of production of TNF-α, IL-2 and IFN-γ and CD107a surface upregulation.

Despite the high level of sequence identity between the human and macaque Ii chain proteins (86%), cross-reactivity to a pool of peptides spanning the human Ii sequence was observed in one rhesus macaque vaccinated with hIi-ME-TRAP. It is important to note that, when this response was mapped to single peptides corresponding to unique human sequences or those shared between human and macaque sequences, an IFN-γ specific response was only observed against peptides unique to human Ii and absent from macaque Ii. Since two recent papers have reported a high frequency of patients with spondyloarthritis have detectable antibodies specific for Ii chain [47], [48] we screened all animals for antibody responses to Ii. Importantly, we did not detect an antibody response to either the human or macaque sequence of Ii chain in any rhesus macaque in this study (Fig. 6). Although we did not immunise macaques with an autologous Ii fusion, no immune responses to mouse Ii were detected in mice immunised with mIi-ME-TRAP (Fig. S2). We therefore conclude that self- tolerance was not broken by vaccinating with an orthologous invariant chain fused to ME-TRAP in this small scale study.

With the discovery of more potent viral vectors and incorporation into heterologous prime-boost regimens, the frequency of antigen specific T cells has increased from tens to thousands of SFC/million PBMCs, yet higher levels are still required. Over the years numerous different approaches have been employed to increase immunity induced by sub-unit vectors, yet many of these fail to translate to clinical studies due to a lack of effect with alternative antigen, vaccines platforms or due to reduced affectivity in higher order species. The ability to augment the response in outbred mice and rhesus macaques in both this study and a chimpanzee Adenoviral based HCV vaccine [49] provides compelling evidence that the Ii invariant chain is a clinically deployable means of improving vaccine immunogenicity in humans.

Since induction of cell-mediated immune responses is not solely a goal of liver stage malaria vaccine development, the increased immune response induced by fusion to Ii observed in this study not only supports the progression of the hIi-ME-TRAP fusion to testing in human clinical trials of its safety, immunogenicity and efficacy, it also suggest that fusion of Ii to other recombinant antigens may be worthy of investigation for development of vectored vaccines for other diseases where cell-mediated immunity may play an important protective role.

## Supporting Information

Figure S1 Flow cytometry gating of murine and macaque samples. a.) *Ex vivo* murine splenocytes or PBMCs were restimulated for 6 hours with the relevant peptides prior to staining for flow cytometry. Gating of antigen specific cytokine producing T cells followed exclusion of dead cells with a FSC vs Live-Dead Aqua gate, removing doublet cells by gating FSC-Area vs FSC-Height and gating on lymphocytes based on size with a FSC-A vs SSC-A gate. Cells were further gated into either CD4^+^CD8^−^ or CD8^+^CD4^−^ before applying IFN-γ^+^, TNF-a^+^ and IL-2^+^ gates. b.) *Ex vivo* rhesus macaque PBMCs were restimulated with the relevant peptides prior to staining for flow cytometry. Gating of antigen specific cytokine producing T cells followed gating on lymphocytes based on size with a FSC-A vs SSC-A gate and excluding doublet cells by gating FSC-Area vs FSC-Height. T cells were identified by gating for CD3^+^ cells, followed by gating into either CD4^+^CD8^−^ or CD8^+^CD4^−^. Single gates were then applied to identify IFN-γ^+^, CD107a^+^, TNF-a^+^ and IL-2^+^ cells.
(TIF)

Figure S2 Response to murine Ii chain in mice. C57BL/6 mice were vaccinated with 10^8^ iu of ChAd63.ME-TRAP or ChAd63.mIi-ME-TRAP and two weeks later the response to TRAP and murine Ii chain measured by flow cytometry. Graphs represent the frequency of IFN-γ^+^ CD4^+^ (left) and CD8^+^ T cell response to TRAP (a.) or murine Ii chain (b.) peptides.
(TIF)

Figure S3 Polyfunctionality of TRAP response following ChAd63-MVA vaccination in mice and rhesus macaques. a.) In the same experiment as described in Fig. 5b, TRAP specific cells were subdivided into cells capable of producing a combination of IFN-γ, TNF-α and IL-2. The proportion of CD4^+^ (top panel) or CD8^+^ (bottom panel) T cells able to simultaneously produce 3 (black), 2 (dark grey) or 1 (light grey) cytokine is indicated by the pie chart, while graphs represent the frequency of CD4^+^ of CD8^+^ T cells producing each possible combination of cytokines. b.) In the same experiment as described in Figure 6, TRAP specific cells from week 9 of the response were subdivided into cells capable of producing a combination of IFN-γ, TNF-α and IL-2 or upregulating degranulation marker CD107a. The proportion of antigen specific CD4^+^ (top panel) or CD8^+^ (bottom panel) T cells positive for 4 (black), 3 (dark grey), 2 (light grey) or 1 (white) functions is indicated by the pie chart, while graphs represent the frequency of CD4^+^ of CD8^+^ T cells producing each possible combination of functions.
(TIF)

Figure S4 **Response of macaque 6021 to TRAP and human Ii chain peptides.** a.) The graph represents the response to TRAP or human Ii chain peptides in rhesus macaque 6021 measured by IFN-γ ELISpot after vaccination. b.) The graph represents the response of macaque 6021 to a single pool of human Ii chain peptide, human Ii peptides present only in the human Ii chain, peptides identical in both the human and macaque Ii chain, macaque peptides only present in the macaque Ii sequence measured at week 15. c.) The graph represent the antibody response in macaque 6021 to human and macaque invariant chain sequence as measured by LIPs assay compared to the positive control mouse anti-human CD74 Clone LN2 (BioLegend). d.) The graphs represent the antibody response in macaque at week 20 to either the human (left) or macaque (right) invariant chain sequence measured by LIPs assay.
(TIF)

Table S1 Ii chain peptide sequences. List the 15-mer peptides (overlapping by 11) of murine Ii chain (NP_034675.1), human Ii chain and peptides unique to macaque Ii chain (XP_001099491.2). Coloured amino acids highlight those which are unique to human (blue) or macaque (red) Ii chain, green coloured amino acid show the common peptides flanking two amino acids not present in the macaque sequence.
(PDF)
