# Supplementary figures and images for: Enhanced Vaccine-Induced CD8+ T Cell Responses to Malaria Antigen ME-TRAP by Fusion to MHC Class II Invariant Chain
Source: PLoS One. 2014 Jun 19;9(6):e100538. doi: 10.1371/journal.pone.0100538 (PMC4063960; doi:10.1371/journal.pone.0100538)

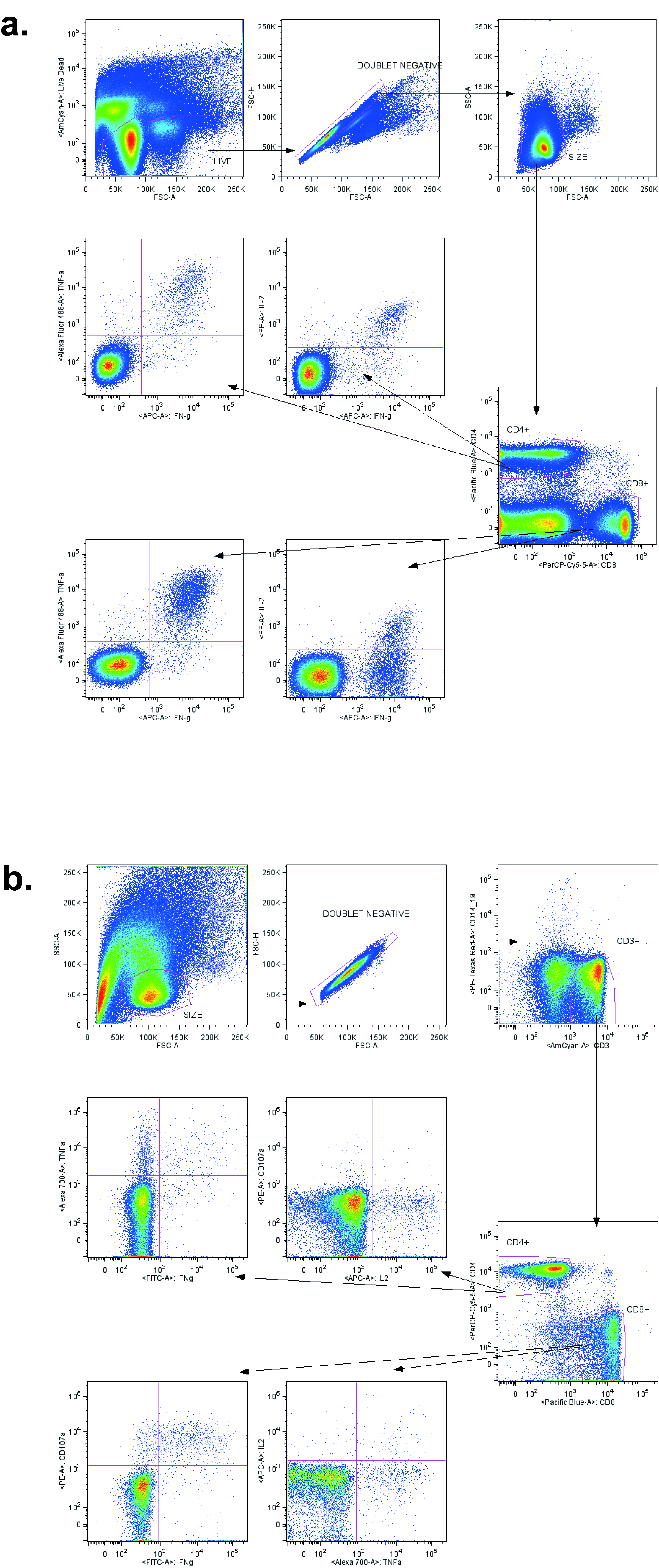

Supplement: Figure S1 — Flow cytometry gating of murine and macaque samples. a.) Ex vivo murine splenocytes or PBMCs were restimulated for 6 hours with the relevant peptides prior to staining for flow cytometry. Gating of antigen specific cytokine producing T cells followed exclusion of dead cells with a FSC vs Live-Dead Aqua gate, removing doublet cells by gating FSC-Area vs FSC-Height and gating on lymphocytes based on size with a FSC-A vs SSC-A gate. Cells were further gated into either CD4+CD8− or CD8+CD4− before applying IFN-γ+, TNF-a+ and IL-2+ gates. b.) Ex vivo rhesus macaque PBMCs were restimulated with the relevant peptides prior to staining for flow cytometry. Gating of antigen specific cytokine producing T cells followed gating on lymphocytes based on size with a FSC-A vs SSC-A gate and excluding doublet cells by gating FSC-Area vs FSC-Height. T cells were identified by gating for CD3+ cells, followed by gating into either CD4+CD8− or CD8+CD4−. Single gates were then applied to identify IFN-γ+, CD107a+, TNF-a+ and IL-2+ cells. (TIF) [file pone.0100538.s001.tif]

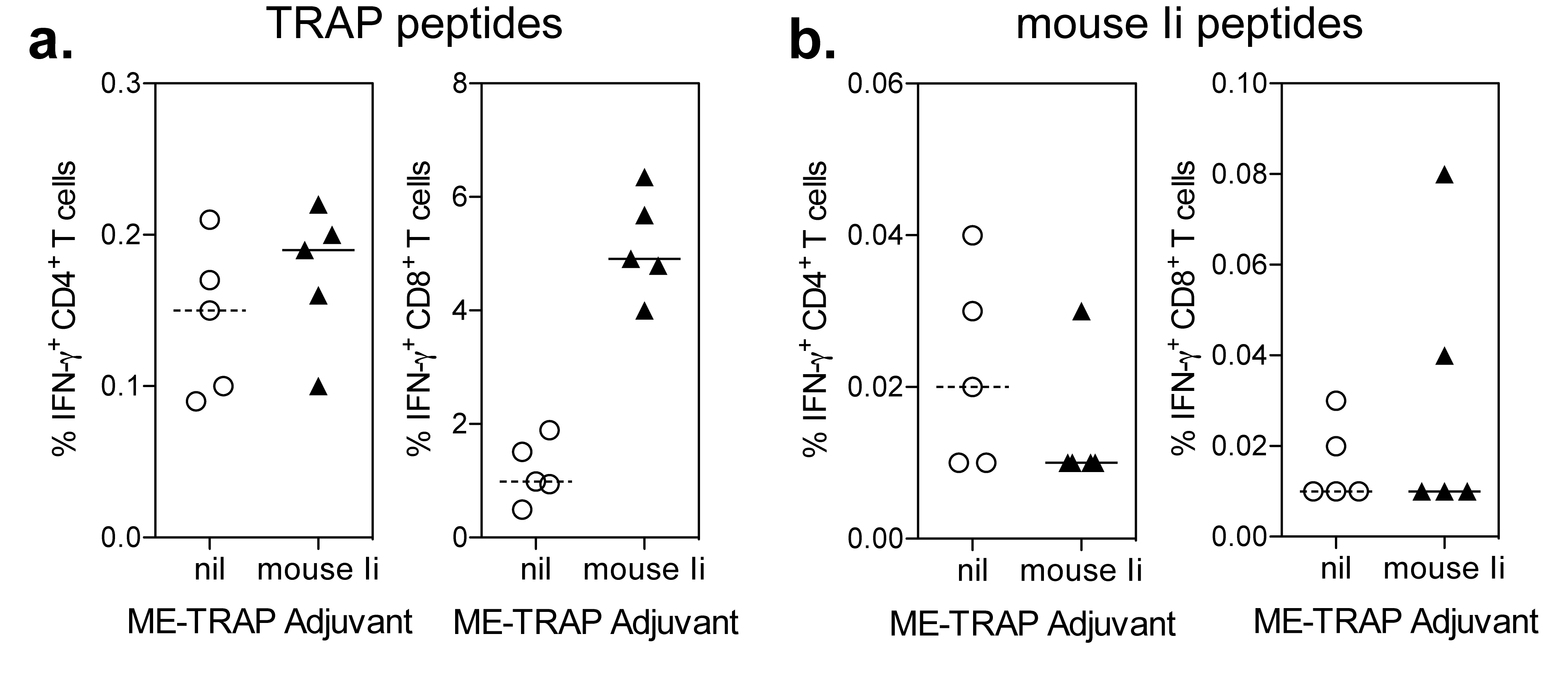

Supplement: Figure S2 — Response to murine Ii chain in mice. C57BL/6 mice were vaccinated with 108 iu of ChAd63.ME-TRAP or ChAd63.mIi-ME-TRAP and two weeks later the response to TRAP and murine Ii chain measured by flow cytometry. Graphs represent the frequency of IFN-γ+ CD4+ (left) and CD8+ T cell response to TRAP (a.) or murine Ii chain (b.) peptides. (TIF) [file pone.0100538.s002.tif]

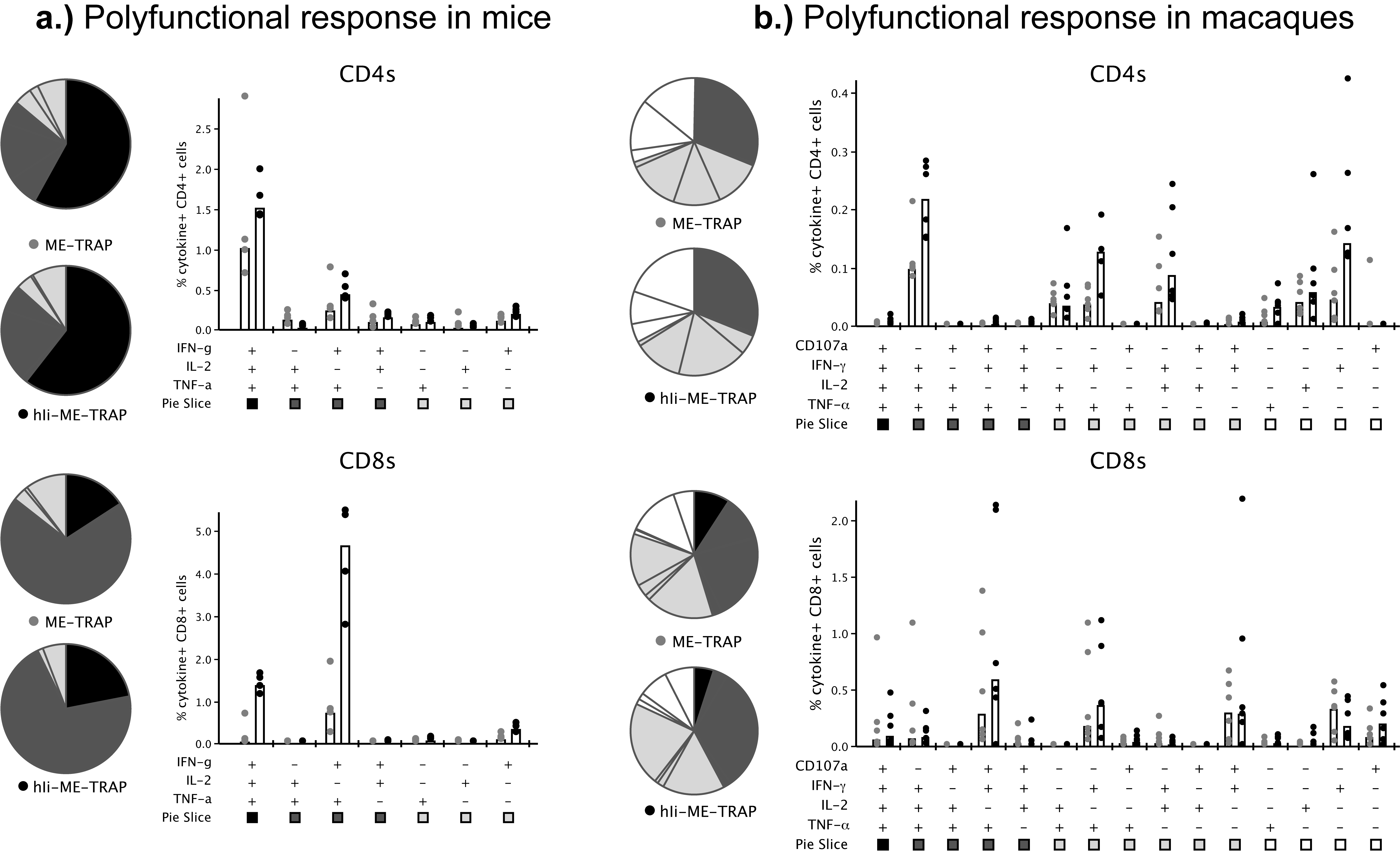

Supplement: Figure S3 — Polyfunctionality of TRAP response following ChAd63-MVA vaccination in mice and rhesus macaques. a.) In the same experiment as described in Fig. 5b, TRAP specific cells were subdivided into cells capable of producing a combination of IFN-γ, TNF-α and IL-2. The proportion of CD4+ (top panel) or CD8+ (bottom panel) T cells able to simultaneously produce 3 (black), 2 (dark grey) or 1 (light grey) cytokine is indicated by the pie chart, while graphs represent the frequency of CD4+ of CD8+ T cells producing each possible combination of cytokines. b.) In the same experiment as described in Figure 6, TRAP specific cells from week 9 of the response were subdivided into cells capable of producing a combination of IFN-γ, TNF-α and IL-2 or upregulating degranulation marker CD107a. The proportion of antigen specific CD4+ (top panel) or CD8+ (bottom panel) T cells positive for 4 (black), 3 (dark grey), 2 (light grey) or 1 (white) functions is indicated by the pie chart, while graphs represent the frequency of CD4+ of CD8+ T cells producing each possible combination of functions. (TIF) [file pone.0100538.s003.tif]

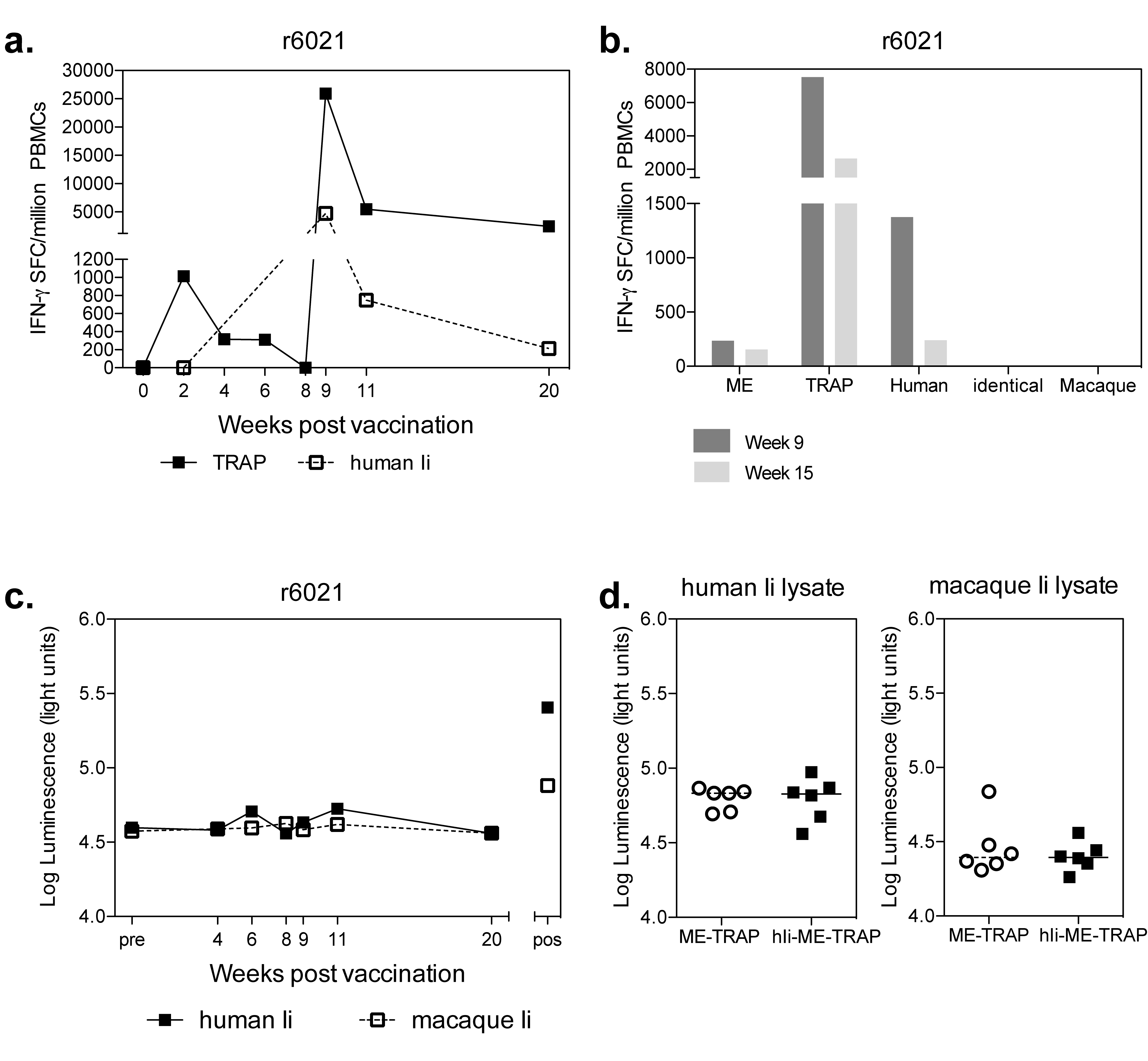

Supplement: Figure S4 — Response of macaque 6021 to TRAP and human Ii chain peptides. a.) The graph represents the response to TRAP or human Ii chain peptides in rhesus macaque 6021 measured by IFN-γ ELISpot after vaccination. b.) The graph represents the response of macaque 6021 to a single pool of human Ii chain peptide, human Ii peptides present only in the human Ii chain, peptides identical in both the human and macaque Ii chain, macaque peptides only present in the macaque Ii sequence measured at week 15. c.) The graph represent the antibody response in macaque 6021 to human and macaque invariant chain sequence as measured by LIPs assay compared to the positive control mouse anti-human CD74 Clone LN2 (BioLegend). d.) The graphs represent the antibody response in macaque at week 20 to either the human (left) or macaque (right) invariant chain sequence measured by LIPs assay. (TIF) [file pone.0100538.s004.tif]
