# Supplementary material for: Enhanced Vaccine-Induced CD8+ T Cell Responses to Malaria Antigen ME-TRAP by Fusion to MHC Class II Invariant Chain
Source: PLoS One. 2014 Jun 19;9(6):e100538. doi: 10.1371/journal.pone.0100538 (PMC4063960; doi:10.1371/journal.pone.0100538)
Supplement: Table S1 — Ii chain peptide sequences. List the 15-mer peptides (overlapping by 11) of murine Ii chain (NP_034675.1), human Ii chain and peptides unique to macaque Ii chain (XP_001099491.2). Coloured amino acids highlight those which are unique to human (blue) or macaque (red) Ii chain, green coloured amino acid show the common peptides flanking two amino acids not present in the macaque sequence. (PDF) [file pone.0100538.s005.pdf]

Mouse NP_034675.1	Human NP_004346.1	Macaque XP_001099491.2
	MHRRRSRSCREDQKPV	MYRSSRRSCQEDQKPV
MDDQRDLISNHEQLP	MDDQRDLISNNEQLP	
RDLISNHEQLPILGN	RDLISNNEQLPMLGR	
SNHEQLPILGNRPRE	SNNEQLPMLGRRPGA	SNNEQLPMLGRRPGT
QLPILGNRPREPERC	QLPMLGRRPGAPESK	QLPMLGRRPGTPESK
LGNRPREPERCSRGA	LGRRPGAPESKCSRG	LGRRPGTPESKCSHG
PREPERCSRGALYTG	PGAPESKCSRGALYT	PGTPESKCSHGALYT
ERCSRGALYTGVSVL	ESKCSRGALYTGFSI	ESKCSHGALYTGFSI
RGALYTGVSVLVALL	SRGALYTGFSILVTL	
YTGVSVLVALLLAGQ	LYTGFSILVTLLLAG	
SVLVALLLAGQATTA	FSILVTLLLAGQATT	
ALLLAGQATTAYFLY	VTLLLAGQATTAYFL	
AGQATTAYFLYQQQG	LAGQATTAYFLYQQQ	
TTAYFLYQQQGRLDK	ATTAYFLYQQQGRLD	
FLYQQQGRLDKLTIT	YFLYQQQGRLDKLTV	
QQGRLDKLTITSQNL	QQQGRLDKLTVTSQN	QQQGRLDKLTVTTQS
LDKLTITSQNLQLES	RLDKLTVTSQNLQLE	RLDKLTVTTQSLQLE
TITSQNLQLESLRMK	LTVTSQNLQLENLRM	LTVTTQSLQLENLRM
QNLQLESLRMKLPKS	SQNLQLENLRMKLPK	TQSLQLENLRMKLPK
LESLRMKLPKSAKPV	QLENLRMKLPKPPKP	
RMKLPKSAKPVSQMR	LRMKLPKPPKPVSKM	
PKSAKPVSQMRMATP	LPKPPKPVSKMRMAT	
KPVSQMRMATPLLMR	PKPVSKMRMATPLLM	
QMRMATPLLMRPMSM	SKMRMATPLLMQALP	
ATPLLMRPMSMDNML	MATPLLMQALPMGAL	MATPLLMQALPMGA
LMRPMSMDNMLLGPV	LLMQALPMGALPQGP	LLMQALPMGAQGP
MSMDNMLLGPVKNVT	ALPMGALPQGPMQNA	ALPMGAQGPMQNA
NMLLGPVKNVTKYGN	GALPQGPMQNATKYG	GAQGPMQNATKYG
GPVKNVTKYGNMTQD	QGPMQNATKYGNMTE	
NVTKYGNMTQDHVMH	QNATKYGNMTEDHVM	
YGNMTQDHVMHLLTR	KYGNMTEDHVMHLLQ	
TQDHVMHLLTRSGPL	MTEDHVMHLLQNADP	
VMHLLTRSGPLEYPQ	HVMHLLQNADPLKVY	
LTRSGPLEYPQLKGT	LLQNADPLKVYPPLK	
GPLEYPQLKGTFPEN	ADPLKVYPPLKGSFP	
YPQLKGTFPENLKHL	KVYPPLKGSFPENLR	
KGTFPENLKHLKNSM	PLKGSFPENLRHLKN	PLKGSFPENLRHLKS
PENLKHLKNSMDGVN	SFPENLRHLKNTMET	SFPENLRHLKSTMET
KHLKNSMDGVNWKIF	NLRHLKNTMETIDWK	NLRHLKSTMETLDWK
NSMDGVNWKIFESWM	LKNTMETIDWKVFES	LKSTMETLDWKVFES
GVNWKIFESWMKQWL	METIDWKVFESWMHH	METLDWKVFESWMHH
KIFESWMKQWLLFEM	DWKVFESWMHHWLLF	
SWMKQWLLFEMSKNS	FESWMHHWLLFEMSR	FESWMHHWLLFEMSK
QWLLFEMSKNSLEEK	MHHWLLFEMSRHSLE	MHHWLLFEMSKHSLE
FEMSKNSLEEKKPTE	LLFEMSRHSLEQKPT	LLFEMSKHSLEQKPT
KNSLEEKKPTEAPPK	MSRHSLEQKPTDAPP	MSKHSLEQKPTEAPP
EEKKPTEAPPKEPLD	SLEQKPTDAPPKESL	SLEQKPTEAPPKVLT
PTEAPPKEPLDMEDL	KPTDAPPKESLELED	KPTEAPPKVLTKCQE
PPKEPLDMEDLSSGL	APPKESLELEDPSSG	APPKVLTKCQEEVSR
PLDMEDLSSGLGVTR	ESLELEDPSSGLGVT	
EDLSSGLGVTRQELG	LEDPSSGLGVTKQDL	
SGLGVTRQELGQVTL	SSGLGVTKQDLGPVP	
	SGLGVTKQDLGPVPM	
